# Genome wide association study of response to interval and continuous exercise training: the Predict-HIIT study

**DOI:** 10.1186/s12929-021-00733-7

**Published:** 2021-05-13

**Authors:** Camilla J. Williams, Zhixiu Li, Nicholas Harvey, Rodney A. Lea, Brendon J. Gurd, Jacob T. Bonafiglia, Ioannis Papadimitriou, Macsue Jacques, Ilaria Croci, Dorthe Stensvold, Ulrik Wisloff, Jenna L. Taylor, Trishan Gajanand, Emily R. Cox, Joyce S. Ramos, Robert G. Fassett, Jonathan P. Little, Monique E. Francois, Christopher M. Hearon, Satyam Sarma, Sylvan L. J. E. Janssen, Emeline M. Van Craenenbroeck, Paul Beckers, Véronique A. Cornelissen, Erin J. Howden, Shelley E. Keating, Xu Yan, David J. Bishop, Anja Bye, Larisa M. Haupt, Lyn R. Griffiths, Kevin J. Ashton, Matthew A. Brown, Luciana Torquati, Nir Eynon, Jeff S. Coombes

**Affiliations:** 1 Centre for Research on Exercise, Physical Activity and Health, School of Human Movement and Nutrition Sciences, University of Queensland, St. Lucia, Brisbane, QLD Australia; 2 Translational Genomics Group, Institute of Health and Biomedical Innovation, Woolloongabba, Brisbane, QLD Australia; 3 Faculty of Health Sciences and Medicine, Bond University, Robina, QLD Australia; 4 Queensland University of Technology (QUT), Centre for Genomics and Personalised Health, Genomics Research Centre, School of Biomedical Sciences, Institute of Health and Biomedical Innovation, Kelvin Grove, Brisbane, QLD Australia; 5 School of Kinesiology and Health Studies, Queen’s University, Kingston, ON Canada; 6 Institute for Health and Sport (iHeS), Victoria University, Melbourne, VIC Australia; 7 Cardiac Exercise Research Group (CERG), Department of Circulation and Medical Imaging, Faculty of Medicine, Norwegian University of Science and Technology, Trondheim, Norway; 8 Caring Futures Institute, SHAPE Research Centre, Exercise Science and Clinical Exercise Physiology, College of Nursing and Health Sciences, Flinders University, Adelaide, SA Australia; 9 School of Health and Exercise Sciences, University of British Columbia, Kelowna, BC Canada; 10 Internal Medicine, Institute for Exercise and Environmental Medicine, University of Texas Southwestern Medical Center, Dallas, TX USA; 11 Department of Physiology, Radboud University Medical Center, Nijmegen, Netherlands; 12 Department of Cardiology, Antwerp University Hospital, Antwerp, Belgium; 13 Department of Rehabilitation Sciences – Research Group for Rehabilitation in Internal Disorders, Catholic University of Leuven, Leuven, Belgium; 14 Baker Heart and Diabetes Institute, Melbourne, VIC Australia; 15 Australia Institute for Musculoskeletal Sciences (AIMSS), Melbourne, VIC Australia; 16 School of Medical and Health Sciences, Edith Cowan University, Joondalup, WA Australia; 17 Department of Cardiology, St. Olavs Hospital, Trondheim, Norway; 18 Guy’s and St Thomas’ NHS Foundation Trust and King’s College London, London, UK; 19 Department of Sport and Health Sciences, University of Exeter, Exeter, UK; 20 Department of Sport, Movement and Health, University of Basel, Basel, Switzerland

**Keywords:** Genetics, V̇O_2_peak training response, Individual variability, GWAS, Polygenic predictor score

## Abstract

**Background:**

Low cardiorespiratory fitness (V̇O_2peak_) is highly associated with chronic disease and mortality from all causes. Whilst exercise training is recommended in health guidelines to improve V̇O_2peak_, there is considerable inter-individual variability in the V̇O_2peak_ response to the same dose of exercise. Understanding how genetic factors contribute to V̇O_2peak_ training response may improve personalisation of exercise programs. The aim of this study was to identify genetic variants that are associated with the magnitude of V̇O_2_peak response following exercise training.

**Methods:**

Participant change in objectively measured V̇O_2_peak from 18 different interventions was obtained from a multi-centre study (Predict-HIIT). A genome-wide association study was completed (n = 507), and a polygenic predictor score (PPS) was developed using alleles from single nucleotide polymorphisms (SNPs) significantly associated (*P* < 1 × 10^–5^) with the magnitude of V̇O_2_peak response. Findings were tested in an independent validation study (n = 39) and compared to previous research.

**Results:**

No variants at the genome-wide significance level were found after adjusting for key covariates (baseline V̇O_2_peak_,_ individual study, principal components which were significantly associated with the trait). A Quantile–Quantile plot indicates there was minor inflation in the study. Twelve novel loci showed a trend of association with V̇O_2_peak response that reached suggestive significance (*P* < 1 × 10^–5^). The strongest association was found near the *membrane associated guanylate kinase, WW and PDZ domain containing 2* (*MAGI2*) gene (rs6959961, *P* = 2.61 × 10^–7^). A PPS created from the 12 lead SNPs was unable to predict V̇O_2_peak response in a tenfold cross validation, or in an independent (n = 39) validation study (*P* > 0.1). Significant correlations were found for beta coefficients of variants in the Predict-HIIT (*P* < 1 × 10^–4^) and the validation study (*P* <  × 10^–6^), indicating that general effects of the loci exist, and that with a higher statistical power, more significant genetic associations may become apparent.

**Conclusions:**

Ongoing research and validation of current and previous findings is needed to determine if genetics does play a large role in V̇O_2_peak response variance, and whether genomic predictors for V̇O_2_peak response trainability can inform evidence-based clinical practice.

*Trial registration* Australian New Zealand Clinical Trials Registry (ANZCTR), Trial Id: ACTRN12618000501246, Date Registered: 06/04/2018, http://www.anzctr.org.au/Trial/Registration/TrialReview.aspx?id=374601&isReview=true.

## Background

Cardiorespiratory fitness (CRF) is measured by peak oxygen uptake (V̇O_2_peak) during a graded exercise test, and is strongly associated with a reduced risk of cardiometabolic diseases and mortality [1]. Improving V̇O_2_peak can generally be achieved by regular endurance exercise training, in a dose-dependent manner [2]. Data typically supports the notion that a higher dose of exercise (volume and intensity) will elicit greater V̇O_2_peak gains [3–7]. Interval training, such as sprint interval training (SIT) and high-intensity interval training (HIIT) have shown comparable [8] and greater [9–13] group mean V̇O_2_peak changes, respectively, compared with moderate-intensity continuous training (MICT). However, there is considerable inter-individual variability in observed V̇O_2_peak improvements following apparently similar exercise training [7, 14]. Identifying the genetic and environmental determinants that can predict exercise response may pave the way to personalised exercise programs that can maximise health outcomes.

An early genome wide association study (GWAS) using data from the HEalth, RIsk factors, exercise Training And GEnetics (HERITAGE) Family Study reported that 21 variants contributed to 49% of the variance in V̇O_2_peak response [15]. However, very few of these variants have been replicated in further testing or other studies suggesting that the variants identified in the HERITAGE study were overfitted to the specific population. In a recent systematic review, we identified 35 studies describing 15 cohorts that found 97 possible variants associated with V̇O_2_peak training response [16]. Only 13 genetic variants were replicated by more than two authors [15, 17–25], and none reached genome-wide significance. A lack of replication and significance in previous research is mostly likely due to underpowered studies that have predominantly been candidate-gene focused [26, 27]. Furthermore, a comparator arm is necessary to discriminate true inter-individual variability from random and technical variability, yet very few studies included such a group, nor did they investigate or control for population stratification. This evidence to-date questions the validity of using currently available commercial genetic tests to prescribe exercise interventions.

Larger sample sizes are needed to build upon current research and to overcome random error in V̇O_2_peak measurement at the individual level. Greater collaboration between research centres using a discovery driven approach free from pre-existing bias is warranted [26]. V̇O_2_peak response between different population groups and training interventions along with assessing how individual factors modulate response, should also be explored [28]. The aim of this study was to use one of the largest cohorts to-date (multi-centre Predict-HIIT [7] study) to complete a GWAS to investigate genetic variants associated with V̇O_2_peak response following exercise training interventions. In addition, we attempted to replicate candidate variants from previous studies, and aimed to build and validate a genetic prediction model for V̇O_2_peak response (polygenic predictor score, PPS) based on the genetic data.

## Methods

### Cohorts

#### Discovery cohort—‘Predict HIIT’

Predict-HIIT participant characteristics, recruiting and study intervention details have been previously outlined [7]. Ethical approval was obtained from the Bellberry ethical committee at the University of Queensland (#2016-02-062-A-1), and from all the institutions involved. Participant data was collated from 18 exercise training interventions across eight universities from three continents. As outlined in our previous paper [7], participant change in objectively measured V̇O_2_peak (indirect calorimetry from a graded exercise test to volitional fatigue on a treadmill or cycle ergometer) was obtained following high-volume HIIT (sessions contained ≥ 15 min of high-intensity efforts in total, n = 225), low-volume HIIT/SIT (sessions contained < 15 min of high-intensity efforts in total, n = 76), or MICT (sessions contained 30 + minutes of continuous exercise at 64–76% maximum heart rate, n = 206). The characteristics of the 507 participants from predominantly European descent used in our GWAS are outlined in Table 1 (24% female, age 55.9 ± 16.9 years, 83% with pathologies and/or elderly). The deoxyribonucleic acid **(**DNA) extraction, preparation and genotyping are outlined below. These details varied based on the study site where the sample was collected, and whether DNA extraction and/or genotyping had already been completed prior to this study. Our quality control measures have limited bias associated with different DNA preparation, extraction methods and genotyping.

**Table 1:** Genome-wide association study participant characteristics. Mean ± standard deviation

	High-volume HIIT	MICT	Low-volume HIIT/SIT	All
Participants	225	206	76	507
Sex (male/female)	187/38	156/50	42/34	385/122
Age (years)	53.4 ± 17.4	61.9 ± 12.2	46.6 ± 20.1	55.9 ± 16.9
Baseline relative V̇O_2peak_ (mL/kg/min)	32.1 ± 11.5	27.6 ± 8.1	30.4 ± 13.7	30.1 ± 10.8
Relative V̇O_2peak_ response (mL/kg/min)	3.4 ± 4.1 Range: − 6.5 to 18.4	2.9 ± 3.6 Range: − 7.4 to 15.3	1.9 ± 2.8 Range: − 4.6 to 8.8	*3.0 ± 3.8 Range: − 7.4 to 18.4
**Number of ‘likely non responders’** (> 1 TEM below + MCID to < 1 TEM below the –MCID)	86 (38%) V̇O_2peak_ response = − 0.6 ± 1.9 (mL/kg/min) PPS: 2.7 ± 2.2	82 (40%) V̇O_2peak_ response = − 0.5 ± 1.9 (mL/kg/min) PPS: 2.5 ± 2.2	42 (55%) V̇O_2peak_ response = − 0.1 ± 1.5 (mL/kg/min) PPS: 2.4 ± 2.6	*210 (41.4%) V̇O_2peak_ response = − 0.43 ± 1.9 (mL/kg/min) PPS: 2.5 ± 2.3
**Number of ‘Likely responders’** (> 1 TEM above the + MCID)	67 (30%) V̇O_2peak_ response = 8.4 ± 2.8 (mL/kg/min) PPS: 6.8 ± 3.7	43 (21%) V̇O_2peak_ response = 8.0 ± 2.1 (mL/kg/min) PPS: 6.3 ± 3.1	12 (16%) V̇O_2peak_ response = 6.4 ± 0.9 (mL/kg/min) PPS: 4.8 ± 2.3	**122 (24.1%) V̇O_2peak_ response = 8.1 ± 2.4 (mL/kg/min) PPS: 6.4 ± 2.7
**Number of ‘uncertain’ responders** (< 1 TEM above to < 1 TEM below + MCID)	72 (32%) V̇O_2peak_ response = 3.6 ± 1.0 (mL/kg/min) PPS: 3.5 ± 2.6	82 (39%) V̇O_2peak_ response = 3.5 ± 3.6 (mL/kg/min) PPS: 3.4 ± 2.9	22 (29%) V̇O_2peak_ response = 3.1 ± 1.0 (mL/kg/min) PPS: 3.5 ± 0.9	175 (34.5%) V̇O_2peak_ response = 3.5 ± 0.99 (mL/kg/min) PPS: 3.5 ± 3.1

TEMs were slightly different for each training intervention and have been outlined in Table 3

Technical error of measurement (TEM) = multiplying mean V̇O_2peak_ value by a previously published coefficient of variation for V̇O_2peak_ of 5.6%, Minimal Clinically Important Difference (MCID) = 3.5 mL/kg/min, Polygenic Predictor Score (PPS). *Significant difference between high-volume HIIT & low-volume HIIT/SIT (*P* < 0.05),

^**^Significant difference between high-volume HIIT, MICT & low-volume HIIT/SIT (*P* < 0.05)

### Validation cohort—‘Improve-HIIT’

For replication of our results, we utilised the unpublished findings from an independent study recently performed in our laboratory (Improve-HIIT). The ‘Improve-HIIT’ study examined the response to high-volume HIIT by randomly allocating 40 sedentary (< 1 h of structured exercise each week) but apparently healthy Caucasian adults (age 18–50) to one of two groups: (i) 6 weeks of supervised high-volume HIIT (5 min warm up, 4 min 90–95% heart rate maximum followed by 3 min recovery repeated 4 times, 3×/week) + prebiotic fibre (oligofructose-enriched inulin) supplementation (12 g/day) or (ii) 6 weeks of supervised high-volume HIIT (3×/week) + placebo (maltodextrin) supplementation (12 g/day). There was no difference in the average V̇O_2_peak response, or the inter-individual variability in V̇O_2_peak response between study groups; as such, this study was deemed appropriate for validating findings from the Predict-HIIT GWAS. Ethical approval was obtained from the Institutional Human Research Ethics Approval committee at the University of Queensland (#2018000398).

Each participant completed a series of tests and several measures were collated before and after the intervention. Tests relevant to this analysis included the completion of an incremental V̇O_2_peak test to exhaustion on a treadmill (Ramped Bruce Protocol) using indirect calorimetry (Parvo Medica True One 2400 System, Parvo Medics, Inc., Sandy, UT, USA) before and after the intervention period, and provision of a saliva sample for genetic analysis (Oragene DNA collection kit, DNA Genotek, Ontario, Canada).

Genotyping, imputation and quality control were completed with the same protocol as for the Predict-HIIT cohort. One sample was removed due to high missing genotyping rate, leaving 39 samples for further analysis. V̇O_2_peak response (post intervention V̇O_2_peak—pre intervention V̇O_2_peak) was calculated for each participant. Fibre/placebo supplement, age, sex, body fat percentage and baseline V̇O_2_peak were not correlated with response and were not included as covariates for analysis. Using PLINK, the top ranked loci (*P* < 1 × 10^−5^) from the Predict-HIIT study were compared in the Improve-HIIT study. Lower ranking loci (*P* < 1 × 10^−4^) were also examined between cohorts (see Table 2 for study characteristics).

**Table 2:** Validation study (Improve-HIIT) participant characteristics**.** Mean ± standard deviation

Intervention	Age (years)	Sex (M = male, F = female)	Baseline V̇O_2peak_ (mL/kg/min)	V̇O_2peak_ response (mL/kg/min)
8 weeks of maltodextrin + 6 weeks of high-volume HIIT	30.4 ± 9.8	4 M, 16 F (20 Total)	29.3 ± 7.4	3.7 ± 4.7
8 weeks of oligo-fructose enriched inulin + 6 weeks of high-volume HIIT	32.8 ± 9.8	5 M, 14 F (19 Total)	35.6 ± 5.3	3.9 ± 5.3
Total	31.6 ± 9.8	9 M, 30 F (39 Total)	32.4 ± 7.1	3.8 ± 5.0

### DNA preparation

#### DNA extraction from whole blood

Genomic DNA from 58 whole blood samples [29] was extracted using a QIAamp DNA blood midi kit (Qiagen, Hilden, Germany) according to the manufacturer’s instructions. The DNA samples were quantified using a Qubit fluorometer 3.0 and all samples were diluted to 100 ng/µL for genotyping.

### DNA extraction from buffy coat

DNA from 93 buffy coats [30] was extracted using a QIAsymphony DSP DNA Mini Kit according to manufacturer’s instructions [31]. The purified genomic DNA was stored at −20 degrees until genotyped.

### DNA extraction from saliva samples

DNA from 289 saliva samples from two studies [32, 33] were extracted using a QIAsymphony (Qiagen) DNA MIDI Kit according to manufacturer’s instructions. The yield and purity were measured using a Trinean DropSense-96. DNA from a further 93 saliva samples from 10 studies [34–43] were extracted using the protocol outlined on the DNA Genotek website [44].

### Genotyping

DNA from 417 samples [29, 30, 32, 34–43] were genotyped using Illumina CoreExome chips 24v1.1 following standard protocols at the Australian Translational Genomics Centre, Princess Alexandra Hospital, Brisbane. A further 116 samples from Norway [33] were genotyped using Illumina CoreExome chips 24v1.2 at the Genomics Core Facility, NTNU. Bead intensity data was processed and normalised for each sample, and genotypes were identified using the Illumina Genome Studio software with corresponding manifest files. SNP coordinates were annotated to the GRCh37 genome build.

### Data quality control

Genotypes at individual SNPs from all cohorts were merged according to the manifest and plink files. Quality control was completed separately on individual cohorts, and included assessment of missingness by individual (threshold < 5%), missingness by genotype (threshold < 5%), Hardy–Weinberg equilibrium in controls (*P* < 1 × 10^−6^), extreme heterozygosity (threshold > 3 standard deviations from mean) and identity by descent threshold at 0.2 of PI_HAT score (n = 13 excluded individuals). For each pair of related samples (PI_HAT > threshold), the sample with the higher missingness rate was removed (n = 3 excluded individuals). Along with quantitative GWAS analysis, we further defined groups based on their relative change in V̇O_2_peak (mL/kg/min) for additional comparisons. Samples were classified as a ‘likely-responder’, ‘likely non-responder/adverse responder’ and ‘uncertain responder’ based on their relative change in V̇O_2_peak (mL/kg/min) following training. As outlined in Williams et al. [7], a likely responder achieved a V̇O_2peak_ response above one minimal clinically important difference (3.5 mL/kg/min) associated with a 10–25% improvement in survival over a 10-year period, plus one technical error of measurement (average baseline V̇O_2_peak multiplied by coefficient variation of 5.6%; calculated for each study). This high threshold for response was used to increase the confidence in the ‘likely responder’/’likely non-responder’ classification. The thresholds are provided in Table 3.

**Table 3:** Thresholds for response

Category	Criteria	High-volume HIIT (mL/kg/min)	MICT (mL/kg/min)	Low-volume HIIT/SIT (mL/kg/min)
Likely responder	> 1 TEM above the + MCID	> 5.3	> 5.0	> 5.2
Likely non-responder	> 1 TEM below + MCID to < 1 TEM below the –MCID	− 5.3 to 1.7	− 5.0 to 2.0	− 5.2 to 1.8
Uncertain responders	< 1 TEM above to < 1 TEM below + MCID	1.7 to 5.3	2.0 to 5.0	1.8 to 5.2

Technical error of measurement (TEM) = multiplying mean V̇O_2peak_ value by a previously published coefficient of variation for V̇O_2peak_ of 5.6%,

Minimal Clinically Important Difference (MCID) = 3.5 mL/kg/min

SNPs with Minor Allele Frequency (MAF) > 0.05 were then used to perform principal component analysis (PCA) for ethnicity identification using SHELLFISH [45]. Ethnic and ancestry outliers (more than 6 standard deviations from the mean on either of the two first principal components (PCs)) were excluded (n = 10). Then, data was imputed with the Haplotype Reference Consortium (HRC) reference panel 1.1 [45] using the Sanger imputation server. SNPs with low imputation quality (INFO score ≤ 0.6) were excluded from further analysis. In total, 26 samples were removed due to large ethnicity deviations from the group, leaving 507 samples for association testing (Table 1). Genomic inflation factor λ and quantile–quantile (Q–Q) plots were used to compare the genome-wide distribution of the test statistic with the expected null distribution. The genomic inflation factor λ is defined as the median of the observed chi-squared test statistic divided by the expected median of the corresponding chi-squared distribution. A λ close to 1 reflects no evidence of inflation, while values up to 1.10 are generally considered acceptable for a GWAS. Baseline V̇O_2_peak, the individual study and PC6 (the 6th principal components from the PCA analysis, which was significantly associated with the phenotype) were included as covariates.

### Statistical analysis

#### V̇O_2_peak response

Normality for V̇O_2_peak was assessed using the Shapiro–Wilk test. An analysis of variance was used to compare average group V̇O_2_peak response between training interventions (high-volume HIIT, MICT, low-volume HIIT/SIT). Variability in response was measured by the range of responses for each intervention. A chi-squared test was used to compare the proportion of likely responders, likely non-responders and those participants classified as uncertain between training groups.

### Association testing of independent V̇O_2peak_ responses

Similar to previous studies in this area [15] investigating polygenic phenotypes (i.e. V̇O_2_peak trainability), we used a quantitative approach rather than a case–control analysis to identify variants associated with V̇O_2_peak response. Association testing was conducted in PLINK [46], using a linear regression. Baseline V̇O_2_peak, the individual study and the PC6 from the principal component analysis were found to be significantly associated with V̇O_2_peak response and were included as covariates in analysis. Age and sex were not associated with the trait. Our findings did not change when age and sex were also included in the association analysis. Thus, we included covariates based on a posteriori instead of a priori knowledge. Association analyses of imputed SNPs was assessed with PLINK best-guess genotypes. Genome-wide significance was set at the standard GWAS threshold of *P* < 5 × 10^−8^ and suggestive significance was set at *P* < 1 × 10^−5^. The single most significant SNP (the lead SNP) was used to represent each of the loci. The cluster plots of the genotyped lead SNP, or supported genotyped SNPs of imputed lead SNP, were checked manually to eliminate poor signals. An analysis of covariance was used to compare the average V̇O_2_peak response for each genotype of the top-ranked SNPs, including baseline V̇O_2_peak, the individual study and PC6 as covariates. Statistical analysis was completed using SPSS (version 23.0, SPSS Inc., Chicago, IL, USA).

### Polygenic predictor score

A polygenic predictor score (PPS) was calculated for each participant using the beta coefficient of the selected SNPs. The PPS was an extension of the ‘summary predictor score’ outlined by Bouchard et al. [15] using data from the HERITAGE study. In our study, we sought to improve on this model to ensure the ‘high response training alleles’/‘effect’ alleles were weighted by the effect size (beta coefficient) derived from our GWAS (see Eq. 1).

1 $$PPS = \mathop \sum \limits_{i}^{k} \beta_{i} *n_{i}$$

where *i* is the index of the SNP in *k* selected SNPs used to calculate the PPS. $\beta_{i}$ is the effect size (beta coefficient of linear regression) of SNP*i* in the PPS model, $n_{i}$ is the number of effect alleles of SNP*i*.

Scores were then added across *k* SNPs to yield a final PPS and a comparison was made between likely responders, likely non-responders and those deemed uncertain (as described earlier). To avoid over-training (inability of model to be generalised to new data), we did a tenfold cross-validation (using MultiBLUP [47]) with the discovery cohort samples and merged the results of 10 test folds for the analysis. The tenfold cross validation was to test the PPS model’s ability to predict V̇O_2_peak response in new data not related to the development of the PPS model internally.

### Replication of candidate loci

The 97 loci identified as candidate loci for V̇O_2_peak response in our recent systematic review [16] were analysed and compared with the top-ranking loci (*α* < 1 × 10^−5^) from the Predict-HIIT study. Lead SNPs from all associated loci were used to calculate the PPS, as well as the 97 genetic variants found previously, were mapped to the nearest gene and submitted as a batch query to the ToppGene pathway analysis software [48]. Biological processes and pathways that appeared in both groups were selected. Genetic variants were also submitted to the GTEx Portal to identify if any SNPs were expressive quantitative trait loci (eQTL) [49].

### Power calculation

Power calculations were performed using the Genome-wide Complex Trait Analysis—Genomic-Relatedness-Based Restricted Maximum Likelihood (GCTA-GREML) calculator [50].

## Results

**Fig. 1:**
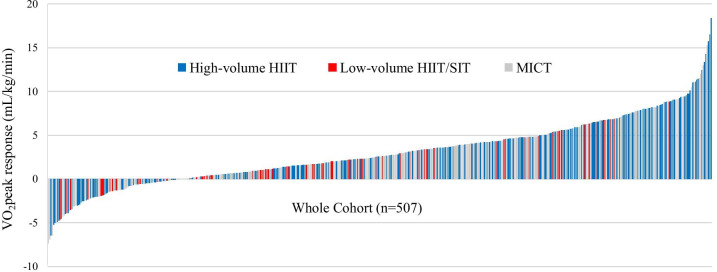
Variability in V̇O_2_peak response between participants included in the GWAS

V̇O_2_peak response was normally distributed. Participants included in the GWAS from high-volume HIIT interventions had a greater V̇O_2_peak response at the group level than participants from a low-volume HIIT/SIT intervention (1.6 mL/kg/min, 95% CI 0.6 to 2.5, *P* = 0.002), but a comparable group V̇O_2_peak response to participants from MICT interventions (0.6 mL/kg/min, 95% CI − 0.1 to 1.3, *P* = 0.1). Participants from MICT and low-volume HIIT/SIT interventions had similar group responses (1.0 mL/kg/min, 95% CI − 0.1 to 2.0, *P* = 0.05). Despite these group mean changes, there was large variability in individual V̇O_2_peak training response within each intervention (see Fig. 1 and Table 1 for ranges). High-volume HIIT had more likely responders than MICT (9%, 0.7 to 17.0, P = 0.03) and low-volume HIIT/SIT (14%, 2.7 to 23.1, P = 0.02). However, high-volume HIIT had similar likely non-responders to MICT (− 2%, 95% CI − 7.2 to 11.1, *P* = 0.7) and less likely non-responders to low-volume HIIT/SIT (− 17%, 95% CI − 4.1 to − 29.3, *P* = 0.01). Furthermore, high-volume HIIT had similar uncertain responders to MICT (− 7%, 95% CI − 2.0 to 15.9, *P* = 0.1) and low-volume HIIT/SIT (3%, − 9.4 to 14.0, *P* = 0.6). To establish the genetic contribution towards this variance in response to each exercise training intervention, we completed a GWAS that was adjusted for significant covariates (individual study that the participant completed, baseline V̇O_2_peak and PC6).

**Fig. 2:**
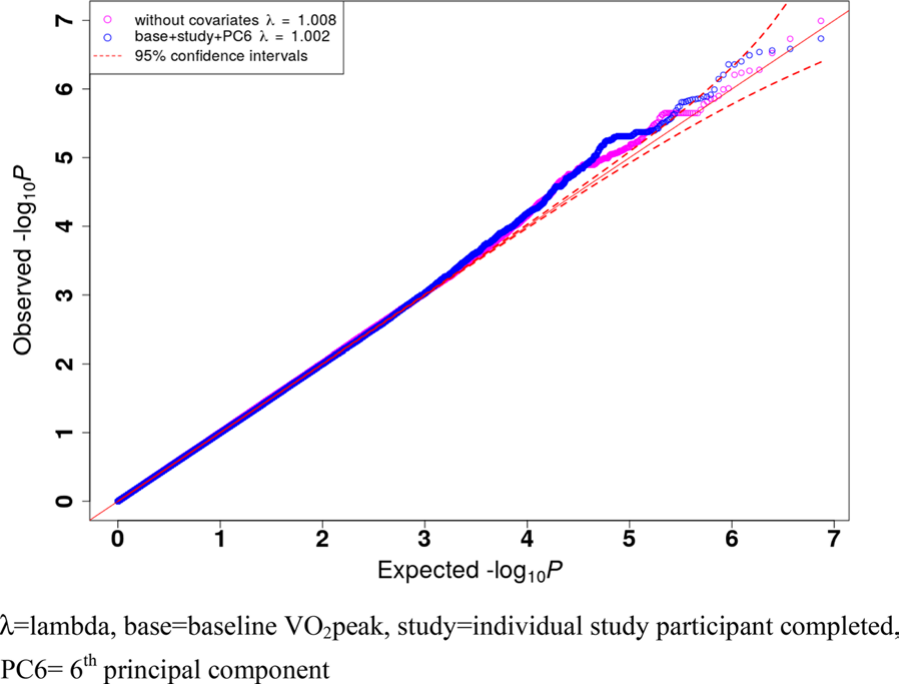
Quantile–Quantile (QQ) plot and genomic inflation factor λ. λ is the observed median of test statistic distribution divided by the expected median of the test statistic distribution. A genomic inflation factor greater than 1.1 indicates there may be some inflation of the GWAS *P*-values; resulting from factors such as population stratification or DNA sample quality. λ = lambda, base = baseline VO_2_peak, study = individual study participant completed, PC6 = 6th principal component

**Fig. 3:**
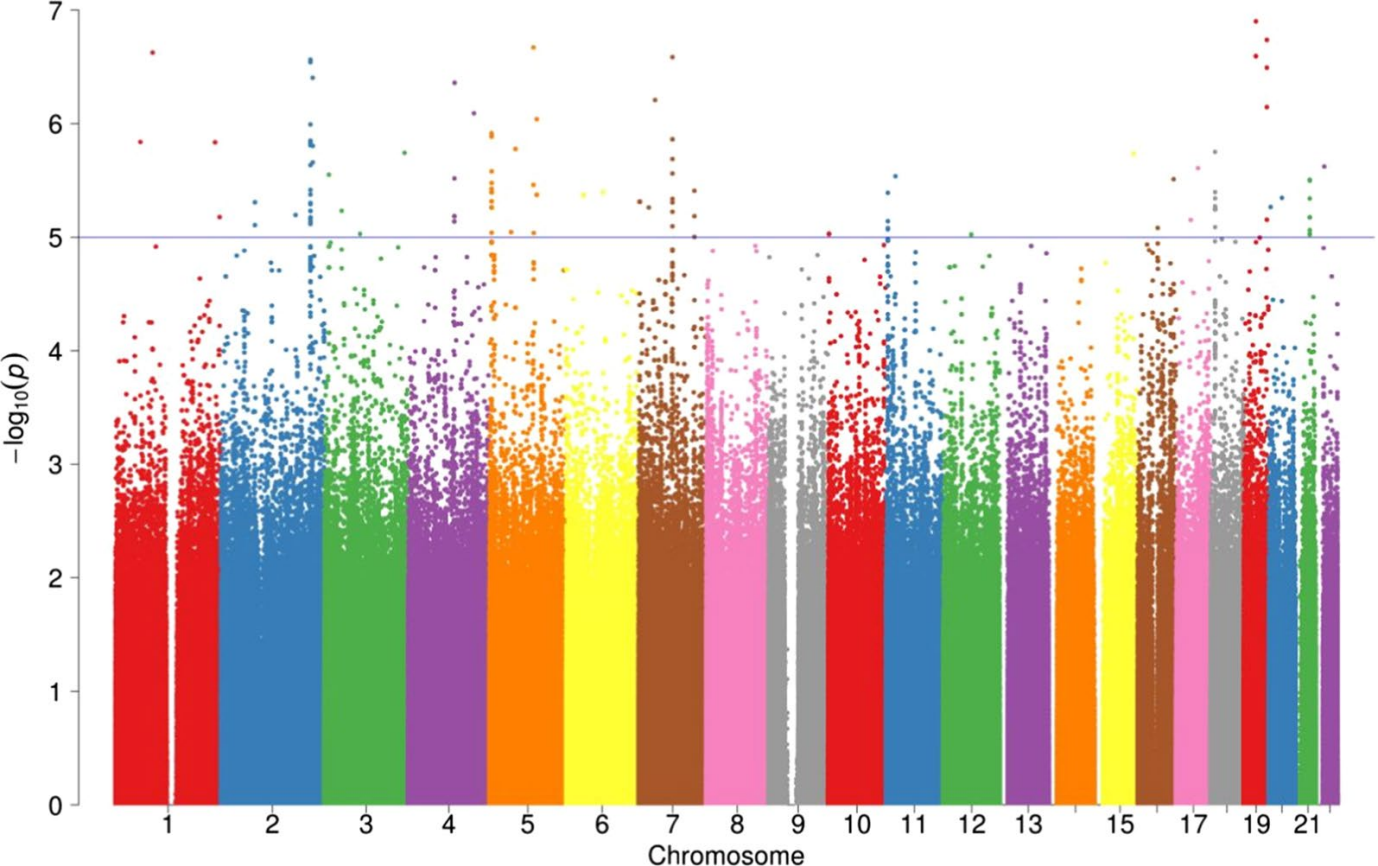
Manhattan Plot of whole Training Cohort. The X-axis represents genomic coordinates, with the negative logarithm of the association p-value for each variant displayed on the Y-axis. Different chromosomes are shown with different colours. The blue line indicates the suggestive significance threshold 1 × 10^–5^

No SNPs reached the typical threshold for genome-wide significance (*P* < 5 × 10^–8^). The Q-Q plot and a genomic inflation factor of 1.002 indicated there was very minor inflation in the study (i.e. population stratification or DNA sample quality), and minor overdispersions of test statistics when compared to the null distribution (Fig. 2). Twelve loci were associated with V̇O_2_peak response at suggestive significance (*P* < 1 × 10^–5^, Fig. 3 and Table 4). The most significant association was found for rs6959961 near the *membrane associated guanylate kinase, WW and PDZ domain containing 2* (*MAGI2*) gene (*P* = 2.61 × 10^–7^). Homozygotes for the response allele (TT, n = 93) had a 2.4 mL/kg/min greater (*P* = 2.8 × 10^–7^) average V̇O_2_peak response than those homozygote for the non-response allele (CC, n = 152) and a 1.3 mL/kg/min greater (*P* = 0.002) average V̇O_2_peak response than heterozygotes (TC, n = 262). The second most significant association (*P* = 2.75 × 10^–7^) was found for rs730747755 near the *Unc-80 Homolog, NALCN Channel Complex Subunit* (*UNC80*) gene. Homozygotes for the response allele (AA, n = 66) had a 2.6 mL/kg/min greater (*P* = 1.2 × 10^–7^) average V̇O_2_peak response than those homozygote for the non-response allele (GG, n = 229), and a 1.8 mL/kg/min greater (*P* = 2.5 × 10^–4^) average V̇O_2_peak response than heterozygotes (AG, n = 212).

**Table 4:** Lead SNP at each locus showing a trend for association with V̇O2peak response

SNP	CHR	BP	P-value	BETA	Closet Gene	Effect allele	Other allele	MAF
rs73074755	2	210627971	2.75 × 10^–7^	1.17	UNC80 *Unc-80 Homolog, NALCN Channel Complex Subunit*	A	G	0.34
rs16875411	5	5359876	1.22 × 10^–6^	− 1.56	ADAMTS16 *A Disintegrin-Like And Metalloprotease (Reprolysin Type) With Thrombospondin Type 1 Motif, 16*	A	G	0.12
rs2236368	6	41309353	4.27 × 10^–6^	1.97	TFEB *Transcription Factor EB*	A	G	0.068
rs111346648	7	2322104	4.86 × 10^–6^	3.27	AMZ1 *Archaelysin Family Metallopeptidase 1*	G	A	0.026
rs6959961	7	79297997	2.61 × 10^–7^	1.19	MAGI2 *Membrane Associated Guanylate Kinase, WW And PDZ Domain Containing 2*	T	C	0.44
rs2657147	11	4573236	4.08 × 10^–6^	− 1.08	OR52M1 *Olfactory Receptor Family 52 Subfamily M Member 1*	G	A	0.37
rs145056992	11	23069615	2.92 × 10^–6^	2.89	CCDC179 *Coiled-Coil Domain Containing 179*	G	A	0.036
rs79687662	15	92921291	1.85 × 10^–6^	2.33	IQGAP1 *IQ Motif Containing GTPase Activating Protein 1*	C	T	0.052
rs11647343	16	84454267	3.10 × 10^–6^	1.12	ATP2C2 *ATPase Secretory Pathway Ca2* + *Transporting 2*	C	A	0.32
rs11874598	18	11064859	4.02 × 10^–6^	1.02	PIEZO2 *Piezo Type Mechanosensitive Ion Channel Component 2*	C	T	0.47
rs149323705	20	30964328	4.52 × 10^–6^	4.42	ASXL1 *Additional Sex Combs Like 1, Transcriptional Regulator*	T	C	0.014
rs73193458	21	33375476	3.13 × 10^–6^	1.49	CLDN14 *Claudin 14*	A	G	0.14

Genome build GRCH37, chromosome (CHR), single nucleotide polymorphism (SNP), physical position (BP), odds ratio (OR), responder/non-responder allele (A1), minor allele frequency (MAF)

**Fig. 4:**
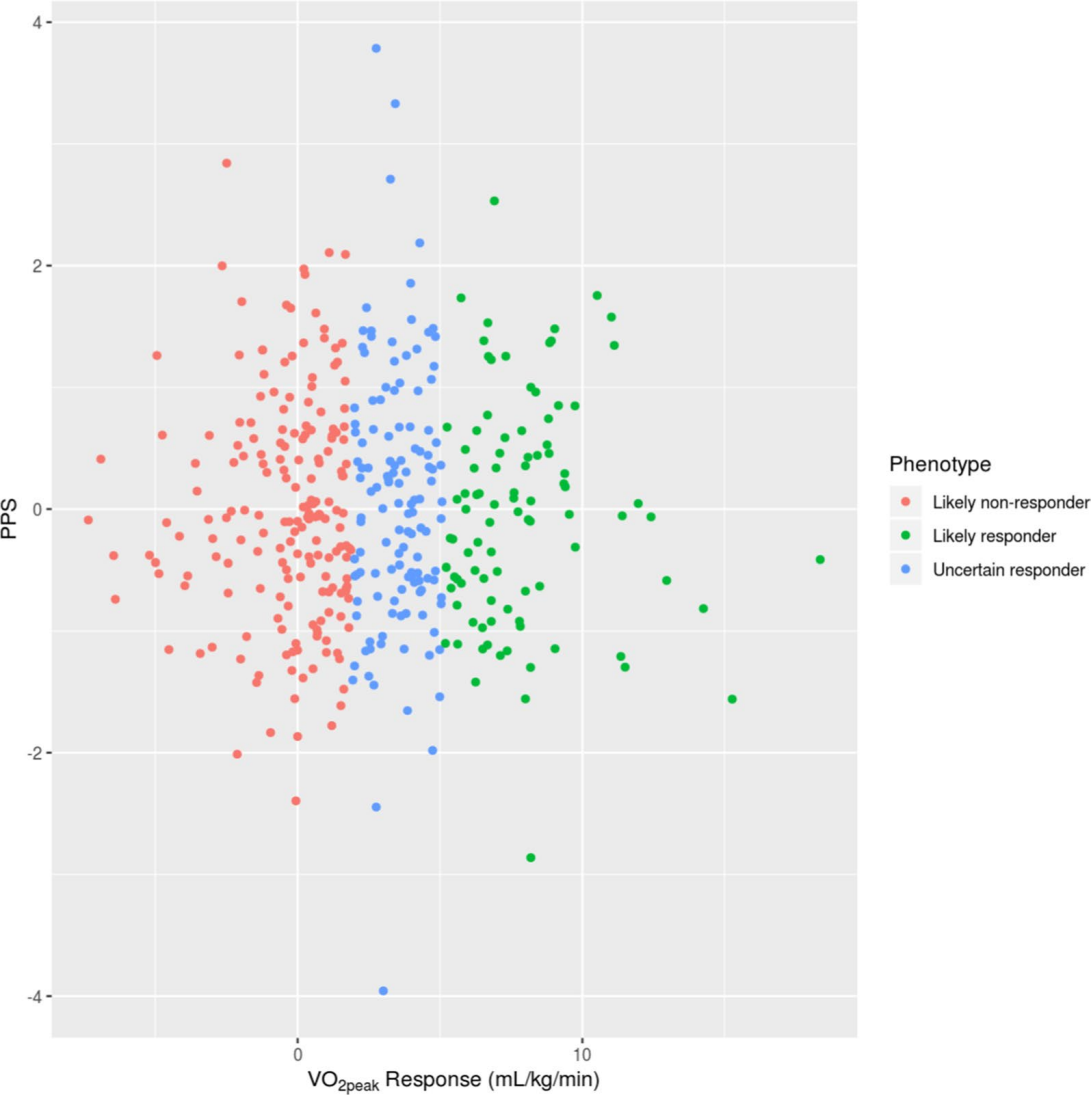
Tenfold cross validation—no correlation between Polygenic Predictor Score (PPS) and V̇O_2_peak response (R^2^ = 0.027, *P* = 0.76). Red, green and blue dots represent likely non-responders, likely responders and uncertain responders, respectively

A tenfold cross validation found the Pearson correlation coefficient between subject polygenic predictor score (PPS) and V̇O_2_peak response (likely responder, likely non responder or uncertain) was not significant (R^2^ = 0.027, *P*-value = 0.76, see Fig. 4). Similarly, the PPS was not able to predict V̇O_2_peak training response in the validation (Improve-HIIT) cohort (R^2^ = 0.001, *P* = 0.8). None of the 12 lead SNPs from our GWAS had a *P-*value < 0.05 in the Improve-HIIT study. Furthermore, from the 992 variants with a *P-*value < 1 × 10^–4^ in the Predict-HIIT cohort, a correlation of beta coefficients in the discovery (Predict-HIIT) cohort and the Improve-HIIT cohort was found to be significant (R^2^ = 0.156, *P*-value = 7.62 × 10^–7^). This suggests these variants in the Improve-HIIT cohort have a significant similar trend of effect as they do in the Predict-HIIT cohort.

Whilst none of the 12 lead SNPs from our GWAS validated SNPs found in previous research, several of our lead 12 SNPs were found near genes that are in similar biological pathways and processes to predictor genes found in previous research (Table 5). Additionally, we were able to validate a number of SNPs from previous research at a nominal level (4 SNPs at *P*-value < 0.05, see Table 6). Furthermore, we found several SNPs to be eQTL in tissues that may influence training adaptations. For example, rs11647343, is an eQTL of *zinc finger DHHC-type palmitoyltransferase 7(ZDHHC7)* in whole blood (*P* = 1.8 × 10^–5^). The SNP, rs2657147, is an eQTL of *tripartite motif containing 68* (*TRIM68*) in subcutaneous adipose tissue (*P* = 4.8 × 10^–8^).

**Table 5:** Gene interactions and common biological pathways and processes between this study and previous findings

Biological pathways	P-value	Genes from input
Signaling events mediated by VEGFR1 and VEGFR2	2.31 × 10^–4^	HIF1A, **IQGAP1**, NRP2, AKT1
Cell adhesion molecules (CAMs)	3.76 × 10^–3^	**CLDN14,** NLGN1, ITGB8 CD6
E-cadherin signaling in the nascent adherens junction	1.05 × 10^–2^	**IQGAP1**, AKT1

Top 10 biological processes	P-value	Genes from input
Ion transport	3.24 × 10^–6^	KCNH8, ENPP3, GRIK4, NLGN1, PPARD, NR3C1, APOE, ACE, RYR2, ACSL1, PIEZO2, SHANK2, KCNQ5, NALCN, KCNF1, SLC22A3, **ATP2C2**, AKT1, PRKG1, GRIN3A, SLC45A1, KCNT1, **UNC80**
Growth	6.25 × 10^–6^	HIF1A, NDN, PPARD, NR3C1, APOE, ACVR1C, TTN, **IQGAP1**, NRP2, **MAGI2**, SHANK2, RPTOR, CD44, AKT1, PRKG1, H19, CNTF
Developmental growth	7.08 × 10^–6^	NDN, PPARD, NR3C1, APOE, ACVR1C, TTN, **IQGAP1**, NRP2, **MAGI2,** SHANK2, AKT1, PRKG1, H19, CNTF
Regulation of cell population proliferation	8.22 × 10^–6^	HIF1A, ENPP3, NDN, BIRC7, PPARD, NR3C1, APOE, ACVR1C, ACE, NRP2, FABP6, PRDM1, GSTP1, **MAGI2**, CD6, RPTOR, CD44, PINX1, AKT1, PRKG1, H19, CNTF
Regulation of membrane potential	8.64 × 10^–6^	KCNH8, GRIK4, NLGN1, RYR2, **PIEZO2,** SHANK2, NALCN, AKT1, GRIN3A, KCNT1, CNTF
Response to oxygen-containing compound	9.78 × 10^–6^	HDAC9, ASXL1, HIF1A, HLCS, ID3, CAT, PPARD, NR3C1, APOE, ACVR1C, ACE, ADCY5, RYR2, **IQGAP1**, PRDM1, ACSL1, GSTP1, CD6, RPTOR, AKT1, GRIN3A
cation transmembrane transport	1.53 × 10^–5^	KCNH8, GRIK4, NLGN1, RYR2, **PIEZO2,** SHANK2, KCNQ5, NALCN, KCNF1, SLC22A3, **ATP2C2,** PRKG1, GRIN3A, SLC45A1, KCNT1, **UNC80**
Cation transport	1.63 × 10^–5^	KCNH8, GRIK4, NLGN1, ACE, RYR2, PIEZO2, SHANK2, KCNQ5, NALCN, KCNF1, SLC22A3, **ATP2C2**, AKT1, PRKG1, GRIN3A, SLC45A1, KCNT1, **UNC80**
Neurogenesis	1.85 × 10^–5^	HDAC9, HIF1A, ID3, NDN, NLGN1, NR3C1, APOE, **IGQAP1**, NRP2, PRDM1, YTHDF1, **MAGI2,** SHANK2, DBX1, CD44, AKT1, PRKG1, GRIN3A, CNTF, ACE, GSTP1
Neuron differentiation	1.93 × 10^–5^	HDAC9, HIF1A, ID3, NDN, NLGN1, NR3C1, APOE, I**GQAP1**, NRP2, PRDM1, YTHDF1, MAGI2, SHANK2, DBX1, CD44, AKT1, PRKG1, GRIN3A, CNTF

Bolded genes from input from the Predict-HIIT cohort. All other genes from previous research

**Table 6:** Findings from previous studies found significant at a nominal level in the Predict HIIT study

SNP	Closest gene	CHR	Beta coefficient	*P*-value in Predict -HIIT cohort	Author	Responder allele/non-responder allele in previous research	Responder allele/non-responder allele in Predict-HIIT study
rs10751308	SHANK2 *SH3 and multiple ankyrin repeat domains 2*	11	0.62	0.02	Gosh et al. [18]	Unknown	T allele (+)
rs10921078	RGS18 *regulator of G protein signaling 18*	1	0.68	0.02	Bouchard et al. [15]	G allele (−)	G (−)
rs1535628	GRIN3A *glutamate ionotropic receptor NMDA type subunit 3A*	9	1.01	0.02	Bouchard et al. [15]	Unknown	A (+)
rs2003298	NLGN1 *neuroligin 1*	2	0.47	0.04	Bouchard et al. [15]	A allele (+)	T (+)

CHR, Chromosome; SNP, single nucleotide polymorphism

The GCTA power calculator found a cohort of 2960 samples would have 80% power to detect a quantitative trait with a true heritability of 30%.

## Discussion

To our knowledge this is one of the largest multi-centre GWAS to investigate CRF response following exercise training. Compared to previous genetic studies in this field of research, we were able to user newer technology and methodologies that increased the validity and accuracy of our results. Across the 507 participants and irrespective of the intervention completed, there was large variability in individual V̇O_2_peak response to high-volume HIIT, MICT and low-volume HIIT/SIT. We were unable to identify genetic variants at a genome-wide significant level that explained this variability in response to each training intervention. However, 12 SNPs were found at a suggestive level of significance and warranted further investigation. Several of our lead SNPs seemed possible candidate genes for predicting V̇O_2_peak response due to their association with previously identified predictor genes, and related biological pathways and processes that may influence training adaptations.

The most significantly associated SNP, *MAGI2,* can influence neuronal cell activin-mediated signalling, and may supress *AKT Serine/Threoine Kinase 1* (*AKT1)* activation [51]. *AKT1* is a V̇O_2_peak response predictor gene identified from previous research, and is one of three genes from the protein kinase B family that can influence growth, differentiation and metabolism [52]. The SNP, rs1130214, found near *AKT1,* was significantly associated (P < 0.05) with V̇O_2_peak response in previous research [52] and was found at a nominal level in our Predict-HIIT cohort (*P* = 0.06). One of our lead SNPs, rs79687662, is found near the *IQ Motif Containing GTPase Activating Protein 1* (*IQGAP1)* gene. *IQGAP1* and *AKT1* genes are both found in the E-cadherin signalling in the nascent adherens junction biological pathway, and together with *Transcription Factor Hypoxia-Inducible Factor-1* (*HIF1A)* and *Neuropilin 2* (*NRP2)* (predictor genes from previous research), are found in the signalling events mediated by the *Vascular Endothelial Growth Factor Receptor 1 (VEGFR1)* and *Vascular Endothelial Growth Factor Receptor 2 (VEGFR2)* biological pathway. Furthermore, a rat model found the catenin (cadherin-associated protein) gene was upregulated in higher responders to HIIT, which helps to regulate angiogenesis, neurogenesis and tissue development [53]. Another recent rodent study found loss of *Iqgap1* may lead to defective *AKT* and *Extracellular Signal-Regulated Kinase 1/2* (*ERK1/2)* signalling and impaired cardiomyocyte hypertrophy [54].

Our second strongest associated lead SNP, rs730747755, is found near the *Unc-80 Homolog, NALCN Channel Complex Subunit* (*UNC80)* gene. *UNC80* is a gene that contributes to a large ion channel complex (the ‘NALCN channelosome”), which includes the *Sodium Leak Channel, Non Selective* (*NALCN)* gene [55]. *NALCN* is a V̇O_2_peak response predictor gene found in previous research [18], and similar to the *UNC80* gene, may influence the resting membrane potential of neuronal cells [55]. There is evidence that genes encoding the NALCN channelosome may contribute to the susceptibility for several diseases, including cardiac diseases, some cancers and psychiatric disorders [56].

Two of our associated lead SNPs were found near genes related to *peroxisome proliferator-activated (PPAR)* activity. The SNP rs14932370 is found near the *ASXL Transcriptional Regulator 1* (*ASXL1)* gene. Overexpression of *ASXL1* may reduce adipogenesis by decreasing *Peroxisome Proliferator-Activated Receptor y* (PPAR*y*) activity [57]. The SNP, rs2236368, is found near the *Transcription Factor EB* (*TFEB)* gene. *TFEB* may regulate mitophagy, and in addition to *Peroxisome Proliferator-Activated Receptor Gamma, Coactivator 1 alpha* (PGC-1α), is considered important for mitochondrial biogenic regulation [58]. *TFEB* may also regulate insulin sensitivity, glucose homeostasis and lipid oxidation [59]. Overexpression of *TFEB* may increase mitochondrial biogenesis and ATP production in skeletal muscle, independently from PCG-1α [59]. A study also found PGC-1α expression can be increased through the dephosphorylation and nuclear translocation of *TFEB* [60]. With these points in mind, a recent study found completing two high-intensity exercise sessions within a short time frame (2 h) increased the nuclear abundance of *TFEB* and the transcription of PCG-1α in 8 healthy young men [61]. Furthermore, overexpression of PGC-1α has been associated with improved V̇O_2_peak at baseline and following endurance training in several studies [62, 63].

Several of our other associated SNPs are found in the same biological processes and pathways to variants identified in previous research [16]. The SNP rs73193458 is found near the *Claudin 14* (*CLDN14)* gene, and together with previously identified V̇O_2peak_ response predictor genes *(Neuroligin 1 (NLGN1), Integrin Subunit Beta 8 (ITGB8)* and *Cluster of Differentiation 6 (CD6))* is involved in the cell adhesion molecules (CAMs) biological pathway. The SNP rs11874598 found near the *Piezo Type Mechanosensitive Ion Channel Component 2* (*PEIZO2)* gene (which is a mechanosensitive ion channel involved in touch, proprioception, and respiratory function [64]); and rs11647343 found near the *ATPase Secretory Pathway Ca2* + *Transporting 2* (*ATP2C2)* gene (which is related to nucleotide binding and calcium transporting ATPase activity and cardiac conduction [64]); along with several other genes identified from previous studies, are involved in cation transmembrane transport biological processes. We also found rs11647343 and rs2657147 to be eQTLs of genes associated with whole blood (*ZDHHC7)* and subcutaneous tissue (*TRIM68*), respectively [49]. In mice, *ZDHHC7* plays a role in *glucose transporter type 4* (*Glut4*) palmitoylation, contributing to glucose homeostasis [65], possibly contributing to metabolic adaptions required for V̇O_2_peak improvements. *TRIM68* variants have been associated with early onset obesity [66] and is upregulated following aerobic exercise [67]. *TRIM68* is associated with ubiquitination [67] and may potentially play a role in proteolytic activity and exercise induced muscle damage. Body composition has been associated with exercise capacity, including maximal workload and oxygen uptake [68, 69]. However, more work is needed to go beyond association and to identify causal variants/genes. Future functional studies are needed.

### Validation

We created a PPS from the top-12 associated loci from the discovery cohort to identify who was more likely to be a responder or non-responder to different forms of training. It was hypothesised that those identified as a lower responder may need a greater training dose than reported in our study to elicit a clinically meaningful response, or other environmental influences may need to be considered. For example, Montero and Lundby [4] have shown non-responders can become responders by increasing the dose of exercise training. Despite many of the suggestively associated SNPs showing a strong connection to previously identified genes, processes and pathways that may influence training adaptions to exercise, there was no significant correlation between the PPS score and V̇O_2_peak response following the tenfold cross-validation. The variants and model could not accurately explain the variance in V̇O_2_peak response or predict who may be a lower or higher responder to each of the training interventions. Likewise, an independent cohort validation, from the Improve-HIIT study, did not support an association of the lead 12 SNPs with variance in V̇O_2_peak response when considered individually, or in the PPS model. This may be due to a relatively small sample size, or in fact that genetics plays a smaller role than previous research has alluded to. Our power calculation found we need at least 2960 samples to detect signals of common variants with a heritability of 30%.

Additionally, we were unable to replicate variants (V̇O_2_peak response predictor genes) identified from previous research [16] at a genome wide or suggestive level of significance. However, we were able to replicate several genetic variants from previous research at a nominal significance level within the Predict-HIIT cohort, including: rs10751308*,* rs10921078*,* rs1535628 and rs2003298 (P < 0.05). Two of these SNPs (rs10921078 and rs2003298) had the same ‘response’ allele in the Predict-HIIT cohort and previous research [15]. These SNPs warrant investigation in future studies. Furthermore, a significant Pearson correlation coefficient was found for the beta coefficient of variants with a *P*-value < 1 × 10^–4^ in the Predict-HIIT cohort the Improve-HIIT cohort. This indicates general effects of the loci (as a group) exist, and a larger sample size may detect many of these effects as statistically significant.

## Limitations

Several limitations may have prevented the finding of more significant associations and validating the proposed PPS model. Firstly, V̇O_2_peak response is considered a complex trait that may result from multiple interactions between genes (epistasis) and epigenetic changes that can affect gene expression [27]. This was made evident with several of the lead Predict-HIIT SNPs sharing common biological pathways and processes to predictor genes identified from previous studies. Larger sample sizes than reported in our study (tens of thousands) are often needed to investigate these gene interactions via a GWAS, and to identify rare variants that may be contributing to overall response [70, 71]. A lack of detail in previous publications prevented some select variants from being replicated. Previous studies have predominantly been candidate-gene focused, and similar to our study, have lacked the necessary statistical power [16]. The validation study also lacked statistical power and the population studied was different to the Predict-HIIT study. The Predict-HIIT study included a mix of healthy, young, older and clinical European population groups from studies with a variety of exercise doses; whereas the validation study was a high volume HIIT intervention on young, healthy but inactive predominantly Caucasian adults, and included a nutrition intervention. Previous studies have predominantly investigated endurance interventions, including participants from a mix of nationalities, and mainly inactive but healthy populations [16]. Moreover, there may be differences in the accuracy of findings between studies based on participant compliance to study protocols. These factors may have influenced the gene expression and the significance of variants discovered in previous research, the validation study and the Predict-HIIT study. If our study had a larger sample size, we could have stratified our analysis to see if associations were different when clustered according to healthy and clinical populations, and training doses. We tried to combat this by including significant covariates in our GWAS model, including the individual study.

Despite limited research, the declining cost of genetic testing has created an abundance of direct-to-consumer (DTC) DNA testing companies [71]. These testing companies often base recommendations on single or very few genes. For example, *Alpha-actinin-3* (*ACTN3)* is a common ‘fitness gene’ found in many DTC tests, whereby consumers are encouraged to modify the intensity, volume or frequency of their exercise training to suit their *ACTN3* genotype. Potential ACTN3 ‘genotype-based training protocols’ for strength and endurance training improvements have been outlined in previous research [72]. For V̇O_2_peak improvement, the authors suggest RR allele homozygotes and RX allele heterozygotes are resistant to muscle damage and better suited to HIIT; whereas XX allele homozygotes have lower skeletal muscle function and poorer recovery, and subsequently are better suited to MICT [72]. Our analysis and other research has shown that exercise-related phenotypes, such as change in V̇O_2_peak response, is a polygenic trait where multiple genes influence various cellular pathways [3, 14] and each gene may contribute only a small percentage to the overall change [16, 26, 73]. We have established that the significance of these genes and associated variants remain uncertain, questioning the importance of genetics in predicting individual response and the validity of commercial tests reliant on limited variants used for personalised exercise prescription.

An even larger study with more participants is needed to advance this field of research. In other areas of genetic research, this is achieved by combining datasets and completing a meta-analysis of many genome-wide association studies. As outlined by Zeggini and Ionnidis [74], combining many GWAS datasets would require a consortium with various institutions and laboratories combining to develop a robust protocol that addresses selection bias, quality control, heterogeneity of populations studied and the replication of biologically plausible previous findings. Based on our findings, we have calculated that at least 2960 participants would need to be included in well-controlled exercise interventions to measure V̇O_2_peak response. Future research should also focus on more than just the genome by using epigenomics, transcriptomics and metagenomics. Having large datasets with this information may help to identify with greater confidence the gene and pathway interactions, and epigenetic changes resulting from environmental influences [75]. Analysis of how exercise dose and quantitative traits including diet, sleep, recovery between training sessions, clinical conditions (e.g. coronary artery disease, type 2 diabetes) and how the microbiome may affect epistasis could also be explored. The Athlome Project Consortium is a collaborative initiative between several institutions to find genetic variants associated with athletic performance [76]. A similar concept could be developed specifically for finding genetic variants associated with V̇O_2_peak response in non-athletes to aerobic training interventions.

## Conclusions

In conclusion, we found 12 novel genetic variants associated with V̇O_2_peak response in the Predict-HIIT study group. These SNPs have common biological pathways and processes to previous research findings_,_ but could not be replicated in a small independent study. Furthermore, cross-validation found the PPS created from the top-associated SNPS did not show significant correlation with whom was likely to be a responder or non-responder to exercise training. Heterogeneity and a lack of power in the discovery (Predict-HIIT) and validation (Improve-HIIT) cohorts may have prevented lead SNPs from being reproduced between studies. Our results highlight the possible risks associated with predictive scores for complex traits. Larger sample sizes with well-prescribed, controlled and accurately measured exercise interventions are required to identify rare variants, gene interactions and epigenetic changes that may influence gene expression and V̇O_2_peak response, and to find the ideal exercise dose to negate non-response. Ongoing research and validation of current and previous findings is needed to confirm if genetics does play a large role in V̇O_2_peak response variance, and whether genomic predictors for V̇O_2_peak response trainability can inform evidence-based clinical practice.

## Data Availability

The datasets used and/or analysed during the current study are available from the corresponding author on reasonable request.

## Abbreviations

ACTN3: Alpha-actinin-3
AKT1: AKT Serine/Threoine Kinase 1
ASXL1: ASXL Transcriptional Regulator 1
ATP2C2: ATPase Secretory Pathway Ca2 + Transporting 2
CAMs: Cell adhesion molecules
CD6: Cluster of Differentiation 6
CLDN14: Claudin 14
DNA: Deoxyribonucleic acid
DTC: Direct to consumer
eQTL: Expression quantitative trait loci
ERK1/2: Extracellular Signal-Regulated Protein Kinase
Glut4: Glucose Transporter Type 4
GCTA-GREML: The Genome-wide Complex Trait Analysis – Genomic-Relatedness-Based Restricted Maximum Likelihood
GWAS: Genome Wide Association Study
HERITAGE: HEalth, RIsk factors, exercise Training and GEnetics
HIF1A: Hypoxia-Inducible Factor 1-Alpha
HIIT: High intensity interval training
IQGAP1: IQ Motif Containing GTPase Activating Protein 1
ITGB8: Integrin Subunit Beta 8
MICT: Moderate intensity continuous training
NALCN: Sodium Leak Channel, Non-Selective
NRP2: Neuropilin 2
PC6: Principal Component Six
PEIZO2: Piezo Type Mechanosensitive Ion Channel Component 2
PGC-1α: Peroxisome proliferator-activated receptor gamma coactivator 1-alpha
PPAR: Peroxisome proliferator-activated receptor
PPARy: Peroxisome proliferator-activated receptor y
PPS: Polygenic predictor score
QQ: Quantile–quantile
SIT: Sprint interval training
SNPs: Single nucleotide polymorphisms
TFEB: Transcription Factor EB
TRIM68: Tripartite Motif Containing 68
UNC80: Unc-80 Homolog, NALCN Channel Complex Subunit
VEGFR1: Vascular Endothelial Growth Factor Receptor-1
VEGFR2: Vascular Endothelial Growth Factor Receptor-2
V̇O2peak: Cardiorespiratory fitness (maximal oxygen uptake)
ZDHHC7: Zinc Finger DHHC-Type Palmitoyltransferase 7

## References

[1] Kodama S, Saito K, Tanaka S, Maki M, Yachi Y, Asumi M (2009). Cardiorespiratory fitness as a quantitative predictor of all-cause mortality and cardiovascular events in healthy men and women: a meta-analysis. JAMA.

[2] WHO. Chronic Diseases and Health Promotion: the World Health Organisation; 2015. http://www.who.int/chp/en/. Accessed 20 January 2019.

[3] Pickering C, Kiely J (2017). Understanding personalized training response: can genetic assessment help?. Open Sports Sci J.

[4] Montero D, Lundby C (2017). Refuting the myth of non-response to exercise training: 'non-responders' do respond to higher dose of training. JAP..

[5] Bonafiglia JT, Rotundo MP, Whittall JP, Scribbans TD, Graham RB, Gurd BJ (2016). Inter-individual variability in the adaptive responses to endurance and sprint interval training: a randomized crossover study. PloS One..

[6] Bacon A, Carter R, Ogle E, Joyner M (2013). VO_2_max trainability and high intensity interval training in humans: a meta-analysis. PloS One..

[7] Williams CJ, Gurd BJ, Bonafiglia JT, Voisin S, Li Z, Harvey N (2019). A multi-center comparison of VO2peak trainability between interval training and moderate intensity continuous training. Front Physiol.

[8] Gist NH, Fedew MV, Dishman RK, Cureton KJ (2014). Sprint interval training effects on aerobic capacity: a systematic review and meta-analysis. J Sports Med.

[9] Boff W, da Silva AM, Farinha JB, Rodrigues-Krause J, Reischak-Oliveira A, Tschiedel B (2019). Superior effects of high-intensity interval vs. moderate-intensity continuous training on endothelial function and cardiorespiratory fitness in patients with type 1 diabetes: a randomized controlled trial. Front Physiol..

[10] Bouaziz W, Malgoyre A, Schmitt E, Lang PO, Vogel T, Kanagaratnam L (2020). Effect of high-intensity interval training and continuous endurance training on peak oxygen uptake among seniors aged 65 or older: a meta-analysis of randomized controlled trials. Int J Clin Pract..

[11] Xie B, Yan X, Cai X, Li J (2017). Effects of high-intensity interval training on aerobic capacity in cardiac patients: a systematic review with meta-analysis. Biomed Res Int.

[12] Ramos J, Dalleck L, Tjonna A, Beetham K, Coombes J (2015). The impact of high-intensity interval training versus moderate-intensity continuous training on Vascular Function: a systematic review and meta-analysis. Sports Med (Auckland, NZ).

[13] Weston K, Wisloff U, Coombes J (2014). High-intensity interval training in patients with lifestyle-induced cardiometabolic disease: a systematic review and meta analysis. Br J Sports Med.

[14] Vellers HL, Kleeberger SR, Lightfoot JT (2018). Inter-individual variation in adaptations to endurance and resistance exercise training: genetic approaches towards understanding a complex phenotype. Mamm Genome.

[15] Bouchard C, Sarzynski M, Rice TK, Kraus WE, Church TS, Sung YJ (2011). Genomic predictors of the maximal oxygen uptake response to standardized exercise training programs. J App Physiol.

[16] Williams CJ, Williams MG, Eynon N, Ashton KJ, Little JP, Wisloff U (2017). Genes to predict VO2max trainability: a systematic review. BMC Genomics.

[17] Defoor J, Vanhees L, Martens K, Matthijs G, Van Vlerken A, Zielinska D (2006). The CAREGENE study: ACE gene I/D polymorphism and effect of physical training on aerobic power in coronary artery disease. Heart.

[18] Ghosh S, Vivar J, Sarzynski M, Sung Y, Timmons J, Bouchard C (2013). Integrative pathway analysis of a genome-wide association study of (V)O(2max) response to exercise training. J Appl Physiol.

[19] Rankinen T, Perusse L, Gagnon J, Chagnon Y, Leon A, Skinner J (2000). Angiotensin-converting enzyme ID polymorphism and fitness phenotype in the HERITAGE Family Study. J Appl Physiol.

[20] Rico-Sanz J, Rankinen T, Joanisse D, Leon A, Skinner J, Wilmore J (2003). Associations between cardiorespiratory responses to exercise and the C34T AMPD1 gene polymorphism in the HERITAGE Family Study. Physiol Genomics.

[21] Rivera M, Dionne A, Fance T, Simoneau J, Perusse L, Chagnon M (1997). Muscle-specific creatine kinase gene polymorphism and VO2max in the HERITAGE Family Study. Med Sci Sports Exerc.

[22] Rivera M, Perusse L, Simoneau J, Gagnon J, Dionne F, Leon A (1999). Linkage between a muscle-specific CK gene marker and VO2max in the HERITAGE Family Study. Med Sci Sports Exerc.

[23] Thomaes T, Thomis M, Onkelinx S, Fagard R, Matthijs G, Buys R (2011). A genetic predisposition score for muscular endophenotypes predicts the increase in aerobic power after training: the CAREGENE study. BMC Genet.

[24] Thompson PD, Tsongalis GJ, Seip RL, Biblie C, Miles M, Zoeller R, Visich P, Gordon P, Angelopoulos TJ, Pescatello L, Bausserman L, Moyna N (2004). Apolipoprotein E genotype and changes in serum lipids and maximal oxygen uptake with exercise training. Metabolism.

[25] Xu Y, Yang H, Ren Z, Yi L (2015). Delta-aminolevulinate synthase 2 polymorphism is associated with maximal oxygen uptake after living-high exercise-high training-low in a male Chinese population. Int J Clin Exp Med.

[26] Eynon N, Voisin S, Lucia A, Wang G, Pitsiladis Y (2017). Preface: genomics and biology of exercise is undergoing a paradigm shift. BMC Genomics.

[27] Sarzynski MA, Ghosh S, Bouchard C (2017). Genomic and transcriptomic predictors of response levels to endurance exercise training. J Physiol.

[28] Bouchard C, Antunes-Correa LM, Ashley EA, Franklin N, Hwang PM, Mattsson CM (2015). Personalized preventive medicine: genetics and the response to regular exercise in preventive interventions. Prog Cardiovasc Dis.

[29] Yan X, Eynon N, Papadimitriou ID, Kuang J, Munson F, Tirosh O (2017). The gene SMART study: method, study design, and preliminary findings. BMC Genomics.

[30] Pattyn N, Vanhees L, Cornelissen VA, Coekelberghs E, De Maeyer C, Goetschalckx K (2016). The long-term effects of a randomised trial comparing aerobic interval training versus continuous training in coronary artery disease: 1-year data from the SAINTEX-CAD study. Eur J Prev Cardiol..

[31] QIAsymphony. QIAsymphony DSP DNA Instructions for Use (Handbook). 2015. file:///C:/Users/camil/Downloads/HB-0977–004_1069185_151035723_R4_HB_QS_DSP_DNA_Kit_0815_WW.pdf. Accessed 20 June 2019.

[32] Taylor JL, Holland DJ, Keating SE, Leveritt MD, Gomersall SR, Rowlands AV (2020). Short-term and long-term feasibility, safety, and efficacy of high-intensity interval training in cardiac rehabilitation: the FITR heart study randomized clinical trial. JAMA Cardiol..

[33] Stensvold D, Viken H, Steinshamn SL, Dalen H, Støylen A, Loennechen JP (2020). Effect of exercise training for five years on all cause mortality in older adults—the Generation 100 study: randomised controlled trial. BMJ..

[34] Bonafiglia JT, Edgett BA, Baechler BL, Nelms MW, Simpson CA, Quadrilatero J (2017). Acute upregulation of PGC-1α mRNA correlates with training-induced increases in SDH activity in human skeletal muscle. Appl Physiol Nutr Metab.

[35] Bonafiglia JT, Rotundo MP, Whittall JP, Scribbans TD, Graham RB, Gurd BJ (2016). Inter-individual variability in the adaptive responses to endurance and sprint interval training: a randomised crossover study. PloS One..

[36] Boyd CJ, Simpson CA, Jung ME, Gurd BJ (2013). Reducing the intensity and volume of interval training diminishes cardiovascular adaptation but not mitochondrial biogenesis in overweight/obese men. PloS One..

[37] Ma JK, Scribbans TD, Edgett BA, Boyd C, Simpson CA, Little JP, et al. Extremely low-volume, high intensity interval training improves exercise capacity and increases mitochondrial protein content in human skeletal muscle. Eur J Mol Integr Physiol. 2013;3(4).

[38] Preobrazenski N, Bonafiglia JT, Nelms MW, Lu S, Robins L, LeBlanc C, et al. Does blood lactate predict the chronic adaptive response to training: a comparison of traditional and talk test prescription methods. Appl Physiol Nutr Metab. 2018. 10.1139/apnm-2018-0343 30058347

[39] Raleigh JP, Giles MD, Scribbans TD, Edgett BA, Sawula LJ, Bonafiglia JT (2016). The impact of work-matched interval training on VO2peak and VO2 kinetics: diminishing returns with increasing intensity. Appl Physiol Nutr Metab..

[40] Scribbans TD, Edgett BA, Vorobei K, MItchell AS, Lioanisse SD, Matusiak JBL (2014). Fibre-specific response to endurance and low-volume high intensity interval training: striking similarities in acute and chronic adaptation. PloS One..

[41] Forbes SC, Sletten N, Durrer C, Myette-Cote E, Candow D, Little JP (2017). Creatine monohydrate supplementation does not augment fitness, performance, or body composition adaptations in response to four weeks of high-intensity interval training in young females. Int J Sport Nut Exer.

[42] Francois M, Pistawka KJ, Halperin FA, Little JP (2017). Cardiovascular benefits of combined interval training and post-exercise nutrition in type 2 diabetes. J Diabetes Complicat.

[43] Ramos JS, Dalleck LC, Borrani F, Mallard AR, Clark B, Keating SE (2016). The effect of different volumes of high-intensity interval training on proinsulin in participants with the metabolic syndrome: randomised trial. Diabetologia.

[44] Genotek. Laboratory protocol for manual purification of DNA from 0.5mL sample. 2019 https://www.dnagenotek.com/ROW/support/protocols/prepIT.html. Accessed 17 May 2019.

[45] Univeristy of Oxford. Shellfish: Parallel PCA and data processing for genome-wide SNP data, UK. 2017. http://www.stats.ox.ac.uk/~davison/software/shellfish/shellfish.php. Accessed 17 May 2019.

[46] COG-Genomics. PLINK 1.90 beta [v.1.90b3.36]. 2020. https://www.cog-genomics.org/plink2. Accessed 10 Novr 2020.

[47] Speed D, Balding DJ (2014). MultiBLUP: improved SNP-based prediction for complex traits. GenomeR.

[48] Division of Biomedical Informatics Cincinnatit Children’s Hospital Medical Center. ToppGene: a one-stop portal for gene list enrichment analysis and candidate gene prioritization based on functional annotations and protein interactions network, USA. 2019. https://toppgene.cchmc.org/. Accessed 13 Nov 2020.

[49] Broad Insititue of MIT and Harvard. The GTEx Portal, USA & UK. 2020. https://gtexportal.org/home/snp/rs73074755. Accessed 10 Nov 2020.

[50] Yang J, Lee Hong S, Goddard ME, Visscher PM (2011). GCTA: a tool for genome-wide complex trait analysis. Am J Hum Genet.

[51] Wu X, Hepner K, Castelino-Prabhu S, Do D, Kaye MB, Yuan XJ (2000). Evidence for regulation of the PTEN tumor suppressor by a membrane-localized multi-PDZ domain containing scaffold protein MAGI-2. Proc Natl Acad Sci USA.

[52] McKenzie J, Witkowski S, Ludlow A, Roth S, Hagberg J (2011). AKT1 G205T genotypeinfluences obesity-related metabolic phenotypes and their responses to aerobic exercise training in older Caucasians. Exp Physiol.

[53] Wisløff U, Bye A, Stølen T, Kemi OJ, Pollott GE, Pande M (2015). Blunted cardiomyocyte remodeling response in exercise-resistant rats. J Am Coll Cardiol.

[54] Sbroggiò M, Carnevale D, Bertero A, Cifelli G, De Blasio E, Mascio G (2011). IQGAP1 regulates ERK1/2 and AKT signalling in the heart and sustains functional remodelling upon pressure overload. Cardiovasc Res.

[55] Bramswig NC, Bertoli-Avella AM, Albrecht B, Al Aqeel AI, Alhashem A, Al-Sannaa N (2018). Genetic variants in components of the NALCN-UNC80-UNC79 ion channel complex cause a broad clinical phenotype (NALCN channelopathies). Hum Genet.

[56] Cochet-Bissuel M, Lory P, Monteil A (2014). The sodium leak channel, NALCN, in health and disease. Front Cell Neurosci.

[57] Park UH, Yoon SK, Park T, Kim EJ, Um SJ (2011). Additional sex comb-like (ASXL) proteins 1 and 2 play opposite roles in adipogenesis via reciprocal regulation of peroxisome proliferator-activated receptor {gamma}. J Biol Chem.

[58] Erlich AT, Brownlee DM, Beyfuss K, Hood DA (2018). Exercise induces TFEB expression and activity in skeletal muscle in a PGC-1α-dependent manner. Am J Physiol.

[59] Mansueto G, Armani A, Viscomi C, D'Orsi L, De Cegli R, Polishchuk EV (2017). Transcription factor EB Controls Metabolic Flexibility During Exercise. Cell Metab.

[60] Theeuwes WF, Gosker HR, Schols A, Langen RCJ, Remels AHV (2020). Regulation of PGC-1α expression by a GSK-3β-TFEB signaling axis in skeletal muscle. Biochim Biophys Acta Mol Cell Res..

[61] Andrade-Souza VA, Ghiarone T, Sansonio A, Santos Silva KA, Tomazini F, Arcoverde L (2020). Exercise twice-a-day potentiates markers of mitochondrial biogenesis in men. FASEB J.

[62] Petr M, Stastny P, Zajac A, Tufano JJ, Maciejewska-Skrendo A (2018). The role of peroxisome proliferator-activated receptors and their transcriptional coactivators gene variations in human trainability: a systematic review. Int J Mol Sci..

[63] Yaghoob Nezhad F, Verbrugge SAJ, Schönfelder M, Becker L, Hrabě de Angelis M, Wackerhage H. Genes whose gain or loss-of-function increases endurance performance in mice: a systematic literature review. Front Physiol. 2019;10(262). 10.3389/fphys.2019.00262 PMC6439621 30967789

[64] Stelzer G, Rosen N, Plaschkes I, Zimmerman S, Twik M, Fishilevich S, et al. The GeneCards Suite: from gene data mining to disease genome sequence analyses. Curr Protoc Bioinformatics. 2016;54(1). 10.1002/cpbi.5 27322403

[65] Du K, Murakami S, Sun Y, Kilpatrick C, Luscher B (2017). DHHC7 palmitoylates Glut4 and regulates Glut4 membrane translocation. J Biol Chem..

[66] Serra-Juhé C, Martos-Moreno GÁ, de Pieri F, Flores R, González JR, Rodríguez-Santiago B (2017). Novel genes involved in severe early-onset obesity revealed by rare copy number and sequence variants. PLoS Genetics..

[67] Dickinson J, DiLugos A, Naymik M, Wolfe A, Curtis D, Huentelman M, Carroll C (2018). Transcriptome response of human skeletal muscle to divergent exercise stimuli. JAP..

[68] Durkalec-Michalski K, Nowaczyk PM, Podgórski T, Kusy K, Osiński W, Jeszka J (2019). Relationship between body composition and the level of aerobic and anaerobic capacity in highly trained male rowers. J Sports Med Phys Fitness.

[69] Radzimiński Ł, Szwarc A, Padrón-Cabo A, Jastrzębski Z (2020). Correlations between body composition, aerobic capacity, speed and distance covered among professional soccer players during official matches. J Sports Med Phys Fitness.

[70] Blanco-Gomez A, Castillo-Lluva S, Del Mar S-F, Hontecillas-Prieto L, Mao JH, Castellanos-Martin A (2016). Missing heritability of complex diseases: enlightenment by genetic variants from intermediate phenotypes. BioEssays : News Rev Mol Cell Dev Biol.

[71] Webborn N, Williams A, McNamee M, Bouchard C, Pitsiladis Y, Ahmetov I (2015). Direct-to-consumer genetic testing for predicting sports performance and talent identification: consensus statement. Br J Sports Med.

[72] Kikuchi N, Nakazato K (2015). Effective utilization of genetic information for athletes and coaches: focus on ACTN3 R577X polymorphism. J Exerc Nutrition Biochem.

[73] MacArther D, North K (2005). Genes and human elite athletic performance. Hum Genet.

[74] Zeggini E, Ioannidis J (2009). Meta-analysis in genome-wide association studies. Pharmocogenomics.

[75] Hoppeler H, Deciphering V (2018). O2, max: limits of the genetic approach. J Exp Biol..

[76] Pitsiladis YP, Tanaka M, Eynon N, Bouchard C, North KN, Williams AG (2016). Athlome Project Consortium: a concerted effort to discover genomic and other "omic" markers of athletic performance. Physiol Genomics.

